# Yucatec Maya Children’s Responding to Emotional Challenge

**DOI:** 10.1007/s42761-023-00205-1

**Published:** 2023-08-14

**Authors:** Shannon M. Brady, Laura A. Shneidman, Cornelio Azarias Chay Cano, Elizabeth L. Davis

**Affiliations:** 1 University of California, Riverside, 900 University Ave, Riverside, CA 92521 USA; 2 Pacific Lutheran University, Tacoma, USA; 3 Colegio de Postgraduados (COLPOS), Texcoco, Mexico

**Keywords:** Display rules, Children, Culture, Emotion, Psychophysiology

## Abstract

While the field of affective science has seen increased interest in and representation of the role of culture in emotion, prior research has disproportionately centered on Western, English-speaking, industrialized, and/or economically developed nations. We investigated the extent to which emotional experiences and responding may be shaped by cultural display rule understanding among Yucatec Maya children, an indigenous population residing in small-scale communities in remote areas of Mexico’s Yucatan peninsula. Data were collected from forty-two 6- and 10-year-old Yucatec children who completed a resting baseline and a structured disappointing gift task. Children were asked about whether specific emotions are better to show or to hide from others and self-reported the intensity of their discrete positive and negative emotional experiences. We observed and coded expressive positive and negative affective behavior during and after the disappointing gift task, and continuously acquired physiological measures of autonomic nervous system function. These multi-method indices of emotional responding enable us to provide a nuanced description of children’s observable and unobservable affective experiences. Results generally indicated that children’s understanding of and adherence to cultural display rules (i.e., to suppress negative emotions but openly show positive ones) was evidenced across indices of emotion, as predicted. The current study is a step toward the future of affective science, which lies in the pursuit of more diverse and equitable representation in study samples, increased use of concurrent multimethod approaches to studying emotion, and increased exploration of how emotional processes develop.

The future of affective science lies in the pursuit of more diverse and equitable representation in study samples, increased use of concurrent multimethod approaches to studying emotion, and increased exploration of how emotional processes develop. There is copious information about the role of culture in *doing emotions* (Mesquita et al., 2017), yet only about 11% of the world’s population (people from nations that are Western, English-speaking, industrialized, and/or economically developed) is well-represented in the published literature (Thalmayer et al., 2021). Norms for emotional responding vary by culture (Matsumoto et al., 2008; Mesquita & Frijda, 1992), and influence children’s developing understanding of others’ and their own emotional experiences. The ways people experience, express, or hide emotions are routed through multiple channels (e.g., facial configurations, tone of voice, body language, physiology, cognition, and more). The goal of this study was to contribute rich multi-method data on emotional responding of children from an underrepresented, majority-world community.^1^

Emotion encompasses subjective experience (i.e., the feeling or sensation one perceives), physiological change (e.g., bodily responses like increases/decreases in stress hormones, muscle tension, heart rate, or respiration), and overt behavior (e.g., facial configurations, body language, tone of voice)—all of which are influenced by cognitive appraisal, interpretation, and meaning-making processes (Frijda, 2008; Lang, 1994; Sroufe, 1996). Dynamic system approaches to emotion (Camras & Witherington, 2005; Thompson, 2011) emphasize the mutual and dynamic interplay of these cognitive, subjective, biological, and behavioral systems. As physiology is thought to underlie overt emotional behaviors and subjective experiences (Brooker & Buss, 2010; Larsen et al., 2008), psychophysiological measures complement behavioral methods by offering insights into internal experiences even when someone is masking outward expression (Calkins & Keane, 2004; Calkins et al., 2007; Gross & Levenson, 1993, 1997; Hinnant et al., 2010). Developmental affective science often assesses autonomic nervous system (ANS) activity (Degnan et al., 2008; Loman & Gunnar, 2010; Quiñones-Camacho & Davis, 2018), including respiratory sinus arrhythmia (RSA) and pre-ejection period (PEP) indices of parasympathetic and sympathetic function, respectively. RSA tends to mark regulatory capacity and skill (Brooker & Buss, 2010). Shorter PEP (i.e., greater sympathetic arousal) has been linked with the experience of negative emotions (except sadness), active task engagement, and increased vigilance (Kreibig et al., 2007; Valenza et al., 2018), whereas longer PEP (i.e., less sympathetic arousal) is associated with positive emotions, such as amusement and joy (Kreibig, 2010).

Emotional experience thus bridges covert and overt channels of responding. The experience of emotion is informed by what kinds of feelings are acceptable to show versus hide through the acquisition of emotional display rules, culturally acceptable forms of emotional expression (and expressive suppression: Saarni, 1984). These display rules contain important information about the kinds of emotional states that are valued or accepted by others in one’s community (Cole & Jacobs, 2018).

Cultural psychology has explored variation in covert and overt channels separately, often focusing on cultural differences in emotional expression. For example, in response to receiving a disappointing gift, European American children overtly displayed more negative affect than Chinese American peers (Garrett-Peters & Fox, 2007), whereas Chinese and Japanese children displayed more neutral expressions (despite self-reporting greater experience of negative emotions) than North American peers (Ip, Miller et al., 2021). When led to believe they broke another’s toy, North American children were more expressive of happiness and sadness than Chinese children (Wang & Barrett, 2014), and Chinese American children displayed lower intensity positive affect than Mexican American children in the context of an unfair social interaction task (Kim et al., 2023).

Others have explored how culture may influence the subjective experience and physiology of emotion. For example, Boiger and colleagues (2018) found differences in the types of anger and shame participants reported experiencing in response to the same situations based on their cultural context (e.g., Belgium, Japan, USA)—indicating cultural variation in what individuals perceive as emotionally evocative, and in what they subjectively experience. With regard to cultural differences in physiology, East Asian participants showed attenuated cardiovascular responses in the context of a stressful task relative to European American participants, by virtue of appraising their nervousness as useful (Yoo et al., 2021). And, East Asian preschoolers experienced increased cortisol in response to *achievement*-related (Chinese participants) and *interpersonal*-related stressors (Japanese participants), while U.S. preschoolers showed *decreased* cortisol responses after these same stressors (Ip, Felt et al., 2021).

## Current Study

We examined an understudied majority-world population to elucidate how cultural display rules shape children’s experience and expression of emotions. The Yucatec Maya are an indigenous population residing in small-scale communities in remote areas of Mexico’s Yucatan peninsula. While almost no prior psychological work has considered the affective functioning of the Yucatec Maya, previous ethnographic work has documented what constitutes appropriate emotional display for Maya adults. In this context, the display of negative emotions is thought to be dangerous. Individuals are expected to hide negative facial expressions to such an extent that another person should be unable to infer their mental/affective state (Hanks, 1993; Le Guen, 2018). In contrast, happiness (ki’imáak óol) is thought to be the base state of normal functioning (Le Guen, 2017). For example, in a task where Maya adults were asked to describe Ekman faces, individuals often labeled smiling faces as “normal” and neutral faces as “happy” indicating that, for adults, a happy or calm state is considered the norm and appropriate for display (Le Guen, 2017). Given societal expectations in this culture where children learn primarily via observation (Gaskins, 1996; Rogoff et al., 1993), open questions remain about how Maya children understand their own emotional experiences.

From a social constructionist perspective, emotions are grounded in the sociocultural context in which they occur (Markus & Hamedani, 2010; Mesquita et al., 2017)—understanding a display rule expectation may have downstream effects on emotional responding. We hypothesized that Maya children would understand that people should hide their negative emotions rather than show them to others, but that this pattern would be more robust for older children. To provide insight into emotional responding, we delineated subjective experience, overt behavior, and physiological responding in the context of a structured, multi-phase emotional challenge. We hypothesized that children would self-report more positive than negative emotion (but younger children would report more negative emotion than would older peers), and that children would display more positive than negative affective behaviors (but younger children would be more expressive).

Regarding physiological responding, as previous work has found SNS arousal to be associated with the experience of negative emotions and mobilization of responses, we hypothesized that children would show shorter PEP during an emotional challenge (Berntson et al., 2007; Dickerson & Kemeny, 2004; Kreibig et al., 2007; Shih et al., 2019; Valenza et al., 2018). On the other hand, dynamic PNS activity (during a task) is thought to index the allocation of regulatory resources in response to a task or stressor (El-Sheikh et al., 2009). Findings have been mixed, however, as to whether patterns of PNS suppression (decreases in RSA resulting in increased heart rate that signify a readiness for behavior in response to threat or challenge; Brooker & Buss, 2010; Porges, 2007) or augmentation (increases in RSA resulting in slowed heart rate and inhibition of sympathetic nervous system input that signify the maintenance of internal equilibrium and support for engagement; El-Sheikh et al., 2009; Obradović & Boyce, 2012) are more indicative of active regulatory efforts. While much research supports that RSA *suppression* in response to challenge is a beneficial pattern of physiological responding (e.g., Beauchaine et al., 2007; El-Sheikh et al., 2011; Gentzler et al., 2009; Porges, 1986; Zeman et al., 2006), other work holds that RSA *augmentation* is more beneficial, indicating active emotion regulatory efforts (Butler et al., 2007; Davis et al., 2017; Hastings et al., 2008). Given these mixed findings, and that this is the first study to explore physiological responding of Yucatec Maya, we had no specific predictions regarding patterns of RSA responding during an emotional challenge, but we predicted that children would show RSA augmentation during a recovery period after that challenge, compared to a resting baseline.

## Method

### Participants

Data collection occurred in a cluster of three Yucatec Maya villages in rural Mexico, near the town of Chemax. Each village had a population ranging from 200 to 600 inhabitants. Families lived in single room structures situated on plots of land that contained one or more nuclear families. Yucatec Maya was the primary language spoken in all villages, although Spanish is the language of instruction in schools and is utilized in the surrounding urban centers. We worked with a native Maya speaker and member of the community (third author) to collect self-report, observational, and psychophysiological data from 42 Yucatec Maya children. Data were collected between 2018 and 2019 in three separate field site visits. The University of California, Riverside institutional review board approved all study procedures for this project [HS#17-272].

Twenty-two children (8 girls, *M*age = 6.72 years, *SD* = 0.29) between ages 6 and 8 years and twenty children (6 girls, *M*age = 10.58 years, *SD* = 0.53) between 9 and 11 years participated. We targeted two separate age groups as Yucatec Maya parents do not consider children to have “begun to start understanding” until two to four years of age, at which point, they are able to consistently follow simple instructions. By the time children reach four to six years of age, they are still considered as “in the process of understanding” and spend the majority of their time interacting with and shadowing older siblings. In fact, children are not considered to have “reached understanding” until about 10 to 12 years of age when they self-initiate chores and perform tasks competently, with no need for supervision (Gaskins, 1996). Thus, our sample was recruited specifically to examine these age-related differences between the younger (~ age 6) and older (~ age 10) age groups as part of our investigation.

All participants were of indigenous Yucatec Maya ancestry and spoke Maya. All participants were also exposed to some Spanish as the result of attending formal schooling, exposure to blends of Spanish and Yucatec Maya spoken in/around the village, and exposure to Spanish-language television programming; however, Maya was the dominant language for all participants and thus the procedure was conducted in Maya. Participants were recruited by word of mouth as the research team traveled throughout the local villages to homes of families with children to invite them to participate in the research study, housed in a makeshift “laboratory” set up in an empty house rented from a local family. Families were identified either by information (obtained from previous field site visits/studies) included in an informal, written database maintained by one of the research team members or through personal acquaintance with the lead experimenter who was native to the primary village. Parental informed consent for children’s participation was provided verbally, after which a day and time was agreed upon for the child to complete the study. Children’s verbal assent was additionally secured before research procedures began.

While household income information was not collected from this sample, the legal minimum wage in Mexico at the times of data collection was ₱88.36/day and ₱102.68/day MXN (in 2018 and 2019, respectively), equivalent to $4.39/day and $5.10/day in USD^2^ (Trading Economics, 2021). The majority of villagers’ economy is based on subsistence farming and other agricultural practices, but men will pursue wage labor positions at nearby tourist locales such as Cancun.^3^ Participating families were compensated with 50 pesos (approximately $2.50 USD) for taking part.

### Procedures

Children individually visited the laboratory, a one-room cinderblock house, to participate. All data collection was conducted by the lead experimenter, a male Maya researcher from the central village, with two US researchers assisting. Usually, children arrived for their appointments alone, but sometimes a parent or sibling would accompany them. The extra family members would typically visit with neighbors in a different building while the child participated, though occasionally the family member preferred to sit in the back of the testing room to observe the study.^4^ The study required a single visit, which lasted approximately 45 min. A curtain was hung to separate the testing space (a small table and chairs, two video cameras) from the staging area where the supporting researchers were located. The study was conducted entirely in Maya, but occasionally the lead experimenter spoke briefly to another researcher in English or Spanish, then translated his speech into Maya for the benefit of the participant. All assessments were audio and video recorded using portable camcorders for later behavioral coding.

At the beginning of the visit, children were given a brief warm-up period to establish rapport with the lead experimenter. After this, children were trained to self-report subjective experiences of discrete emotions (sadness, anger, fear, embarrassment, happiness) and their intensity using schematic face-based rating scales. This self-reported affective information was collected at five different time points (described below).

Next, they participated in a “prize rank” task wherein they ranked five toys (e.g., light-up ring, bouncy ball, glow-in-the-dark lizard, stretchy frog, parachute man, teething ring) in order of preference from their “most favorite” to their “least favorite” and were told they would receive their most favorite prize later. The five toys presented were not identical for each child, but combinations always included the teething ring, light-up ring, and glow-in-the-dark lizard as these were often ranked as the least- (teething ring) and most- (light-up ring, lizard) favorite prizes.

This was followed by the application of cardiac acquisition apparatus (7 self-adhesive electrodes/leads placed on the participants’ rib cages and collar bones) that transmitted continuous psychophysiological data to an ambulatory device that locally recorded ECG and ICG signals. The lead experimenter introduced the sticky electrodes to the child by placing one on the back of his hand and allowing the child to touch it or put it on their own hand. A second experimenter (the last author) completed electrode placement and data previewing while the lead experimenter explained that children would wear the sticky sensors on their bodies so that the experimenters could listen to their hearts during the study. Next, children sat quietly at the table for approximately two minutes, which provided a measure of their physiological resting baseline.

After the baseline phase, the child engaged in various tasks on their own (e.g., completing a puzzle) or with the experimenter (e.g., answering interview questions about emotional experience). At the end of the visit, children were thanked and given a small prize and the honorarium for participation.

#### Gift Task

Approximately 30 min into the study, children participated in an adapted “disappointing gift” task (Cole, 1986; Saarni, 1984) which was divided into two phases.

##### Disappointment Phase

Children were given an envelope containing a prize (presumably their highest-ranked toy). Instead, when they opened it, they were surprised to find their lowest-ranked toy, which was additionally broken beyond use (e.g., if it was a bouncy ball, it had been cut in half). Children were left alone with this disappointing gift for approximately one minute (the lead experimenter moved behind the curtain with the other researchers).

##### Resolution Phase

Next, the lead experimenter returned, feigned dismay at having given the wrong gift by mistake and corrected the error by providing the child with their most-desired (intact) prize. This phase lasted approximately 30–45 s. This multi-phase disappointment task thus presented children with an emotionally challenging experience and a recovery phase, throughout which we assessed both psychophysiological and behavioral responding.

### Measures/Materials

#### Self-Report

##### Endorsements of Whether Discrete Emotions Are Better to Show or Hide

To assess children’s understanding of their culture’s emotional display rules regarding the suppression of negative emotion, we collected children’s self-reported endorsement of whether five discrete emotion categories (sadness, anger, fear, embarrassment, and happiness) were better to show to others or whether it was better they be hidden. Specifically, children were asked, “When you feel [sad], what do you think? Is it better to show that you are sad or is it better to hide it so that other people can’t see it?” Children’s responses were dichotomously coded as endorsing either “show” or “hide.”

##### Subjective Experience

To assess children’s subjective experience of emotion, we employed schematic face-based rating scales depicting varying degrees of the same five discrete emotions they were previously asked about. The experimenter trained children on what each level of the scale meant by pointing at each face one by one and explaining that it meant feeling *not at all (ma’)*, *a little bit (jun p’íit)*, *more (mas)*, or *very (jach)* much of the emotion. After introducing the scale levels for one emotion, the experimenter asked children to point to a certain face (e.g., the one that is “a *little bit*” sad?) to verify that the child understood how to self-report using the scales. If the child did not answer correctly, the experimenter relabeled the face the child pointed at (e.g., “this one is *more* sad”) and would repeat the explanation until the child responded correctly to the prompt. After the comprehension check for each emotion, the experimenter moved on to the next discrete emotion. Using these scales, we assessed whether the child felt not at all, a little bit, pretty much, or very [sad (*triiste*^5^), angry (*p'uja'an*), afraid (*sa’jáak’*), embarrassed (*su'ulak*), happy (*ki'imak wóol*)] at five different time points throughout the visit, with the child providing an intensity rating for each of the five emotions at each time point. These measurements occurred at the following timepoints: (1) after the prize rank, (2) after the resting baseline post-electrode placement, (3) after the administration of an autobiographical emotion interview, (4) after a puzzle task, and (5) after the gift task.^6^

#### Overt Behavior

For the purposes of quantifying children’s affective behaviors, we developed a novel coding scheme. To establish the scheme, two research assistants and the first author watched video recordings of disappointment and/or resolution phases of the gift task to note any observable affective behaviors (e.g., smiling, frowning, deep sighing; described below). Once the research team created an exhaustive list of behaviors, these were reduced into two sets of codes to determine degree of positive and negative affective behavior on a zero to two scale (0 = no positive/negative affective behavior, 1 = some positive/negative affective behavior, 2 = much positive/negative affective behavior). Children’s expressive behavior was coded for affective behaviors rather than discrete emotional expressions (such as happiness, sadness, or anger), because the feelings evoked by the initial disappointment could have been experienced and expressed as anger, sadness, or both (Shih et al., 2018), and the feelings evoked by the resolution phase could have been experienced and expressed as relief, joy, or embarrassment.

Research assistants completed extensive training until they reached a 90% agreement rate with the primary coder (the first author), after which they were able to code videos independently. Each case was double-coded. Video recordings were coded in 10-s intervals during the portion of the task when children were left alone with the gift. To assign a code for each 10-s interval, coders assessed the duration and intensity of children’s affective behaviors over the approximately 60-s phase. Inter‐rater reliability was assessed using a one-way, absolute agreement, average-measures intraclass correlation (ICC) to assess the degree to which coders agreed upon scores for each interval for 100% of the cases (Hallgren, 2012; McGraw & Wong, 1996). The resulting ICCs for negative affective behavior (ICC = .92) and for positive affective behavior (ICC = .93) were excellent (Cicchetti, 1994). Discrepancies were resolved through discussion between the coders and first author (primary coder).

##### Negative Affective Behavior

Indicators of negative affect included verbalized complaints (e.g., “it’s broken; I don’t want this one”), facial (e.g., pouting, frowning) or bodily expressions of negative emotion (pushing the toy/envelope away from the body, throwing the gift, sighing, sucking teeth, and crying). Each 10-s interval was assigned a code of 0 (no negative affective behavior), 1 (some negative affective behavior), or 2 (much negative affective behavior). Distinctions between levels (“some” and “much”) were based on the duration of children's expressions of these behaviors. For example, a facial expression of pouting for less than 5 s (half of the interval) was coded as a 1, whereas pouting through most of an interval (more than 5 s) was coded as a 2. Other bodily behaviors were evaluated based on intensity—for example, sucking teeth and sighing were coded as a 1 (some negative affect), whereas behaviors such as pushing the toy away, crying, screaming, throwing the object, or throwing the head back in frustration were coded as a 2 (much negative affect). Once any behavior within a single interval received a score of 2, the interval was scored as a 2, even if it also contained more mild negative affective expression behaviors.

##### Positive Affective Behavior

Positive affective behavior codes were assigned primarily based on the duration of expressive behavior within each interval. Codes of 0 (no positive affective behavior) were assigned if children quietly manipulated the prize/envelope in which it came, stared at the prize, or stared elsewhere with no smiling, laughing, or otherwise animated expression. Indicators of positive affect included playing with the toy in an enjoyable manner, facial or verbal expressions of positive emotion (e.g., laughing, smiling), and visible physical excitement (e.g., swinging legs in one’s chair, anticipatory fidgeting). For example, smiling for less than 5 s (half of the interval) was coded as a 1 (some positive affect), whereas smiling for the majority of a 10-s interval was coded as a 2 (much positive affect). Again, once any behavior in a single interval received a score of 2, it was scored as such.

We computed proportion scores to index the extent of expressed negative and positive affective behaviors. These were calculated by dividing the number of intervals in which a child exhibited any affective behavior (i.e., received a score of 1 or 2) by the number of usable (full-length, codable) intervals. Proportion scores closer to 1.0 indicate more extensive affective behavior (a greater number of 10-s intervals in which a child displayed any affective behavior).

#### Physiological Response

Physiological data, including electrocardiogram (ECG), and impedance cardiography (ICG) were collected throughout the study, but of interest here were data from three distinct episodes: (1) a resting baseline toward the beginning of the visit in which children sat quietly by themselves for two minutes, (2) the disappointment phase of the gift task in which children were alone with the wrong, broken toy, and (3) the resolution phase of the gift task, after children had been provided with the correct toy. ECG and ICG were locally recorded to the ambulatory device (MindWare Technologies, Westerville, OH, USA). Data were collected via self‐adhesive electrodes placed on the participants’ rib cages and collar bones (Bar-Haim et al., 2000; Porges & Byrne, 1992; Sherwood et al., 1990). Three electrodes were placed on the children’s distal right collarbone, lower left rib, and lower right rib to acquire an electrocardiograph (ECG) signal. Four additional electrodes were placed to acquire impedance data (ICG). Two voltage electrodes were placed below the suprasternal notch and xiphoid process, and two current electrodes were placed on the back with one 3–4 cm above and one 3–4 cm below the voltage electrodes. Electrode leads were connected to an ambulatory monitor secured in a small bag placed on the back of the child’s chair (de Geus et al., 1995; Willemsen et al., 1996). Once electrodes were placed on the torso, experimenters measured the distance between the two electrodes located at the top and bottom of the sternum (for later computation of pre-ejection period, the index of sympathetic activation). After data signals were previewed and participants indicated they were comfortable in their seat, physiological recording began for the resting baseline measure (2 min).

##### Cardiac Physiology Processing and Scoring

The cardiac data were processed off-line using MindWare’s Heart Rate Variability (HRV 3.2) and Impedance Cardiography (IMP 3.2) analysis applications.

##### RSA

Respiratory sinus arrhythmia (RSA) was the measure of parasympathetic activity. RSA spectral power was integrated over a high‐frequency bandpass range set at .15–.80 Hz and calculated in 30-s epochs. This range was derived from estimates of the average respiration rates (i.e., typically between 16 and 25 breaths per minute) of children in middle childhood (Johnson et al., 2017; Quiñones‐Camacho & Davis, 2018).^7^ Each 30-s epoch was visually inspected for errors (most often these were missed R-waves or peaks misidentified as R-waves), which were manually corrected as needed. Higher RSA values indicate increased variability in heart rate at the frequency of respiration—characterized by increased shortening and lengthening of heart periods in a phase relationship with inspiration and expiration, respectively (Berntson et al., 1993).

##### PEP

Pre-ejection period (PEP), a measure of contractility of the heart, was used as an index of sympathetic activity. PEP was derived from the ECG and ICG signals. Impedance data were ensemble averaged within 30-s epochs, and each waveform was visually inspected and edited as needed. Using the method outlined by Berntson et al. (2004), PEP was qualified as the time interval in milliseconds from the onset of the Q‐wave to the B point of the dZ/dt wave. The Q‐onset in the ECG was placed using a validated automated scoring algorithm. Waveforms were visually inspected to ensure accurate placement and adjusted if needed. PEP was averaged across all 30-s epochs for each subject. Higher PEP indicates a longer time between contraction of the left ventricle and opening of the aortic valve (Hinnant et al., 2010; Sherwood, 1993); longer PEP times indicate decreased sympathetic nervous system activity (Kreibig et al., 2007).

To calculate RSA and PEP scores, we averaged across the available full 30-s epochs to derive an episode average for each participant when at rest (baseline) and during each phase of the disappointing task (disappointment, resolution). Higher average values indicate greater parasympathetic activity (higher RSA) and less sympathetic activity (longer PEP).

## Results

This results section is organized into five parts. First, we describe preliminary analyses, including missing data, descriptive statistics, and bivariate correlations among study variables. We then present primary analyses to evaluate our hypotheses about children’s adherence to their culture's display rules across self-report, observational, and physiological measures, as well as potential age-related differences.

### Preliminary Analyses

#### Post hoc Power Analysis

A post hoc power analysis was conducted using G*Power version 3.1.9.6 (Faul et al., 2009) for power estimation, based on our current sample size (*N* = 42) in an ANOVA with a between-within interaction for 2 groups and 5 repeated measures. With significance criterion set at α = .05 and correlation among repeated measures of .10, the estimated power achieved to detect a small to medium effect size (.25) is .87. Thus, the obtained sample size of *N* = 42 is adequate to test the study hypotheses.

#### Missing Data

Data were partially missing for only one participant; this child did not have usable physiological data due to electrodes coming loose. Analyses make use of all available data, so degrees of freedom vary.

#### Descriptive Statistics and Correlations

Descriptive statistics and correlations are presented separately for younger (*n* = 22; Table 1) and older (*n* = 20; Table 2) children. Among the younger participants, girls (coded as 1) showed more positive affective behavior during the disappointment phase of the gift task (*r* = .33, *p* = .03) than boys (coded as 0). Otherwise, there were no other significant associations with child sex, *rs* < .24, *ps* > .13. Similarly, among the older age group, there were no significant associations between child sex and any of the other variables of interest (*rs* < .37, *ps* > .11). Given that we had no specific hypotheses about sex, it is not considered further.

**Table 1:** Means, standard deviations, and correlations for younger (6- to 8-year-old) children (*n* = 22)

Variable	*M*	*SD*	1	2	3	4	5	6	7	8	9	10	11	12	13	14	15
1. Child Sex	0.36	0.49															
2. Baseline RSA	6.77	1.09	−0.21														
3. Baseline PEP	74.36	9.24	−0.06	0.35													
4. Disappointment RSA	6.73	1.12	−0.10	0.80**	0.50*												
5. Disappointment PEP	78.11	9.25	0.13	0.20	0.91**	0.38											
6. Resolution RSA	6.36	1.25	−0.20	0.80**	0.30	0.76**	0.28										
7. Resolution PEP	77.91	9.90	−0.11	0.22	0.70**	0.11	0.73**	0.28									
8. Disappointment NAB	0.16	0.27	0.24	−0.69**	−0.27	−0.60**	−0.32	−0.68**	−0.35								
9. Disappointment PAB	0.11	0.23	0.60**	−0.11	0.02	−0.01	0.10	− 0.25	0.06	−0.05							
10. Resolution NAB	0.06	0.22	−0.09	0.47*	0.09	0.46*	−0.07	0.43*	−0.29	− 0.13	−0.14						
11. Resolution PAB	0.36	0.39	0.18	−0 .10	0.15	−0 .05	0.14	−0 .15	0.18	−0 .09	0.48*	−0 .26					
12. Avg. SR Sadness	0.39	0.77	−0 .17	−0.08	0.07	−.15	0.14	−0.13	0.02	−0.08	−0.24	0.03	0.11				
13. Avg. SR Anger	0.02	0.06	0.09	0.15	−0.03	0.06	0.08	0.27	−0.10	−0.09	−0.16	0.10	−0.30	0.00			
14. Avg. SR Fear	0.09	0.21	0.22	−0.15	−0.34	0.10	− 0.33	−0.10	−0.51*	0.36	− 0.06	0.09	− 0.07	−0.09	−0.14		
15. Avg. SR Embarrass	0.08	0.24	0.22	−0.02	0.21	0.08	0.37	−0.03	0.18	−0.15	0.56**	−0.09	0.17	−0.03	0.29	−0.04	
16. Avg. SR Happiness	1.81	1.21	0.03	0.43*	0.27	0.19	0.26	0.20	0.33	−0.50*	0.30	0.18	0.24	0.28	0.00	−0.56**	0.06

*M *and *SD* are used to represent mean and standard deviation, respectively. For the variable of Child Sex, boys were coded as 0 and girls were coded as 1

*RSA* respiratory sinus arrhythmia, *PEP* pre-ejection period, *NAB* negative affective behavior, *PAB* positive affective behavior, *SR* self-reported

^*^p < .05; **p < .01

**Table 2:** Means, standard deviations, and correlations for older (9- to 11-year-old) children (*n* = 20)

Variable	*M*	*SD*	1	2	3	4	5	6	7	8	9	10	11	12	13	14	15
1. Child Sex	0.30	0.47															
2. Baseline RSA	7.58	1.12	−0.08														
3. Baseline PEP	77.51	10.38	0.32	0.02													
4. Disappointment RSA	7.42	1.08	−0.12	0.89**	−0.10												
5. Disappointment PEP	77.89	9.99	0.25	−0.07	0.92**	−0.20											
6. Resolution RSA	7.30	1.21	−0.19	0.88**	−0.10	0.77**	−0.21										
7. Resolution PEP	76.95	11.36	0.35	−0.32	0.84**	−0.38	0.83**	−0.31									
8. Disappointment NAB	0.02	0.06	0.37	−0.08	0.33	−0.08	0.25	−0.09	0.38								
9. Disappointment PAB	0.10	0.16	−0.10	− 0.30	−0.28	−0.07	−0.36	− 0.14	−0.15	−0.07							
10. Resolution NAB	0.03	00.10	0.25	− 0.18	0.01	−0.06	0.13	−0.25	0.13	0.75**	−0.04						
11. Resolution PAB	0.65	0.27	− 0.08	− 0.39	− 0.08	− 0.35	− 0.18	−0.15	−0.05	0.07	0.17	−0.06					
12. Avg. SR Sadness	0.08	0.18	− 0.30	0.33	0.15	0.17	0.18	−0.06	− 0.23	−0.19	−0.24	−0.15	−0.42				
13. Avg. SR Anger	0.02	0.06	0.15	0.03	0.29	0.26	0.25	−0.11	0.22	−0.14	−0.03	−0.10	0.02	0.04			
14. Avg. SR Fear	0.11	0.23	−0.13	−0.12	0.21	−0.14	0.13	−0.25	0.18	−0.21	0.38	− 0.15	0.10	0.14	0.28		
15. Avg. SR Embarrass	0.20	0.32	−0 .14	0.19	− 0.10	0.00	−0.05	−0.06	−0.11	−0.27	−0.06	−0.20	0.01	0.30	−0.11	0.20	
16. Avg. SR Happiness	2.45	0.75	0.25	−0.21	0.03	−0.02	−0.15	−0.18	−0.04	0.08	0.29	−0.08	0.32	−0.17	0.07	−0.11	−0.11

*M* and *SD* are used to represent mean and standard deviation, respectively. For the variable of Child Sex, boys were coded as 0 and girls were coded as 1

*RSA* respiratory sinus arrhythmia, *PEP* pre-ejection period, *NAB* negative affective behavior, *PAB* positive affective behavior, *SR* self-reported

^*^p < .05; **p < .01

For both age groups, baseline RSA was positively correlated with RSA during both the disappointment and resolution phases of the gift task (*r*s < .80, *p*s < .001), and baseline PEP was positively correlated with PEP at both phases of the gift task (*r*s < .70, *p*s < .001). The correlations between disappointment phase RSA and resolution phase RSA, and between disappointment phase PEP and resolution phase PEP were also significant (*r*s < .73, *p*s < .001). Fisher’s *z* tests showed that the magnitudes of these correlations were comparable in both age groups (*z*s < 1.06, *p*s > .29).

Among older children, the proportion of negative affective behaviors exhibited during the disappointment phase was significantly correlated with the proportion of negative affective behaviors exhibited during the resolution phase (*r* = .75, *p* < .001) and this association was significantly stronger than the same correlation for younger children (*r* =  − .10), Fischer’s *z* = 3.31, *p* < .001. No other significant correlations between variables emerged for the older age group.

Uniquely for the younger children, RSA during the disappointment phase was correlated with negative affective behavior during the disappointment (*r* =  − .60, *p* = .003), and resolution (*r* = .46, *p* = .03) phases. Positive affective behavior during the disappointment and resolution phases was also correlated (*r* = .48, *p* = .02). Per Fisher’s *z* tests, however, these correlations were not statistically significantly different in magnitude from the same (non-significant) correlations for the older age group (*z*s =  − 1.05, *p*s > .05). Both baseline (*r* =  − .69, *p* < .001) and resolution (*r* =  − .68, *p* < .001) phase RSA were negatively correlated with negative affective behavior during the disappointment phase. In contrast, baseline RSA (*r* = .47, *p* = .03) and resolution (*r* = .43, *p* = .046) phase RSA were positively correlated with negative affective behavior during the resolution phase. When compared to the parallel correlations among the older age group, these latter correlations were significantly different (*z*s >  − 2.07, *p*s < .04).

In sum, the pattern of physiology showed relative stability in both RSA and PEP across the baseline, disappointing, and resolution phases. For behavior, in contrast, older children showed stronger consistency in their negative affective behaviors across the two phases of the gift task than did younger children. This may point to developmental differences in the extent to which children have internalized and can adhere to display rules about when and whether different emotions ought to be shown versus hidden from others.

### Discrete Emotion Show/Hide Endorsements

**Fig. 1:**
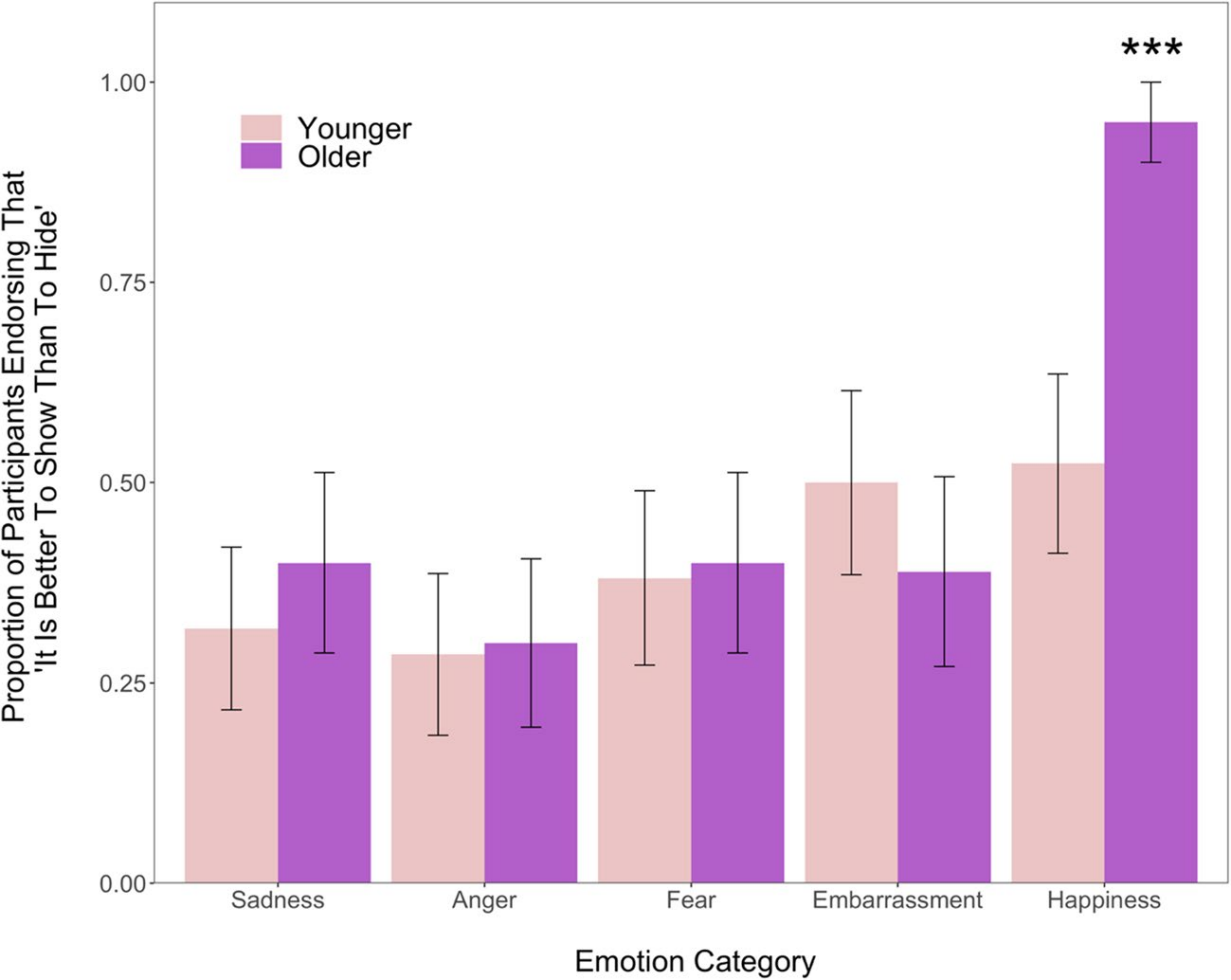
Proportion of participants who endorsed it being better to “show” (versus “hide”) the five specific discrete emotions. Significantly more older children endorsed happiness as being better to show than younger children and significantly more older children also endorsed happiness as “better to show to others” than all negative emotions. Error bars indicate standard error. ***ps < .01

To examine children’s understanding of their culture’s display rules about whether it is better to show or hide certain emotions, we conducted Bonferroni-corrected single sample *t*-tests for each age group separately (see Fig. 1). These indicated that for younger children, the proportion of show/hide endorsements for sadness, anger, fear, embarrassment, and happiness were not significantly different from chance (*t*s < 2.12, *p*s > .47). Among the older children, however, participants endorsed happiness as better to show to others (versus hide) at greater than chance levels, *t*(19) = 9.00, *p* < .001, *d* = 2.01, 95% CI [0.96, 2.01].

We also found that, among the older age group, happiness was endorsed as better to show to a significantly greater extent than were anger, embarrassment, fear, and sadness (*t*s > 4.37, *p*s < .001, *d*s > .90). There were no significant differences in comparisons between discrete emotions among the younger children (*t*s < 1.58, *p*s > .12, *d*s < .49). There were also no differences in the endorsement of whether the negative emotions (anger, embarrassment, fear, and sadness) were better to show or better to hide. Older children, however, endorsed happiness as better to show significantly more than did younger children, *t*(27.70) =  − 3.48, *p* = .008, *d* =  − 1.08, 95% CI [− 1.83, −0 .50]. Our hypotheses about children’s understanding of cultural display rules were thus partially supported; children among both age groups generally indicated that negative emotions (sadness, anger, fear, and embarrassment) should be hidden from others rather than shown openly. Regarding positive emotions, older children were more likely to report that happiness was better to show than younger children.

### Subjective Experience

Because we did not have emotion self-reports interleaved with each phase of the gift task, we considered children’s emotional experience across the five assessments (after the prize rank, the resting baseline, the autobiographical emotion interview, the puzzle task, and the gift task) to index children’s willingness to report emotional experience at the broader level, across a *series* of challenging tasks and novel situations.

To probe patterns in subjective experience, we ran a mixed factorial ANOVA with emotion intensity ratings entered as the dependent variable, age group (younger versus older) as the between-subjects factor, and two within-subjects factors [the five discrete emotions (sadness, anger, fear, embarrassment, happiness), and timepoint (the five self-report ratings)]. We found a significant main effect of discrete emotion, *F*(1.76; 70.48) = 115.412, *p* < .001, *η*^2^ = .63, but no main effects of age group or rating timepoint (*F*s < 1.45, *p*s > .23).

**Fig. 2:**
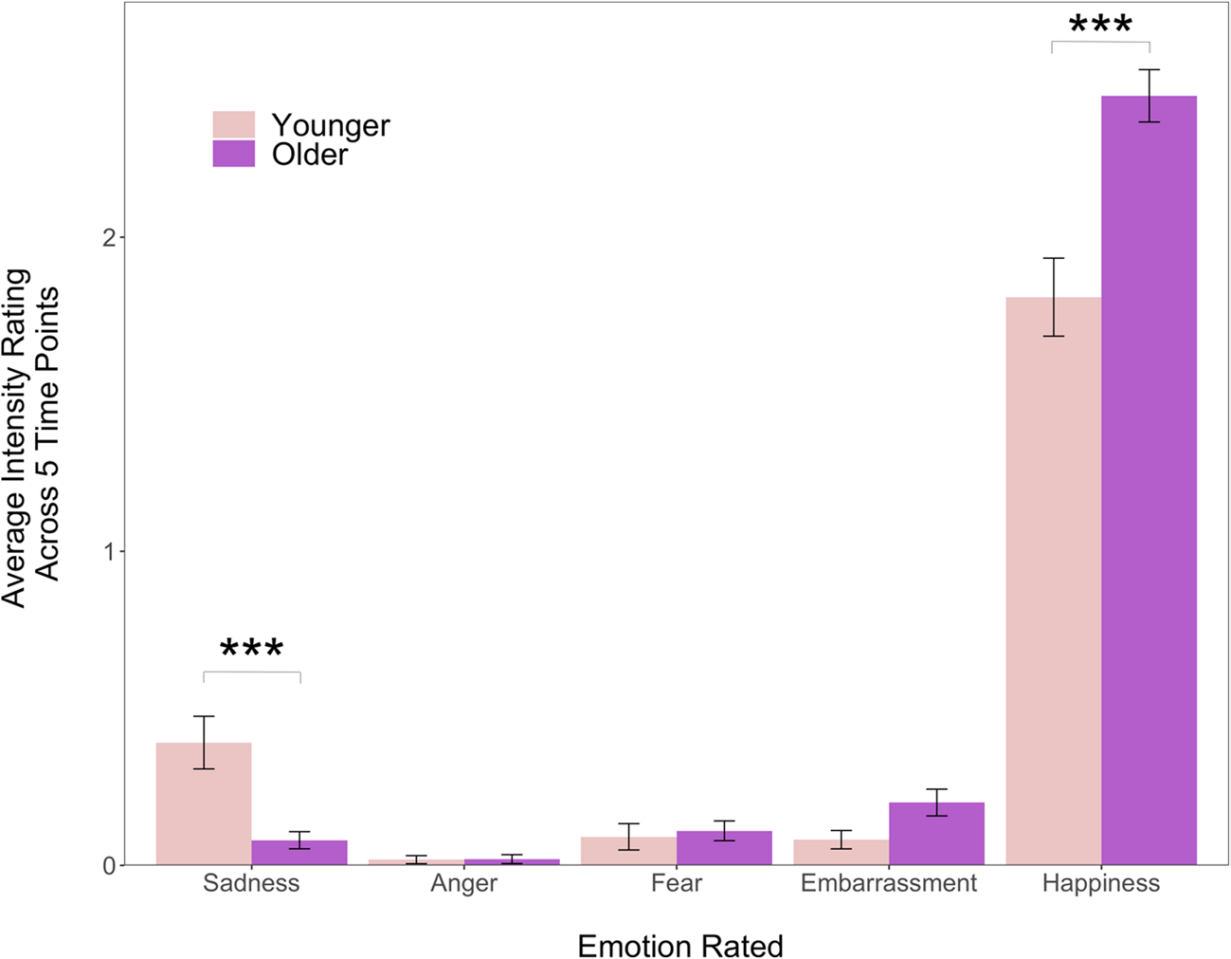
The average self-report rating of emotional intensity for the five discrete emotions, assessed at five different time points during the study. Error bars indicate standard error. Children from both age groups reported more experience of happiness than negative emotions (*p* < .001), but older children self-reported experiencing more happiness and less sadness than younger children. ***ps < .01

As shown in Fig. 2, while there were no significant interactions between age group and timepoint or emotion and timepoint, there was a significant interaction between age group and discrete emotion, *F*(1.76, 70.48) = 4.25, *p* = .02, *η*^2^ = .06. Examination of the simple effect of age group showed age differences in self-reported happiness, *F*(1, 208) = 17.6, *p* < .001, *η*^2^ = .08, and sadness, *F*(1, 208) = 11.5, *p* = .004, *η*^2^ = .05, but not anger, fear, or embarrassment (*F*s < .13, *p*s > .11). Older children self-reported experiencing more happiness (*M* = 2.45, *SD* = 0.83) than younger children (*M* = 1.81, *SD* = 1.30), and less sadness (*M* =0 .08, *SD* = 0.27) than younger children (*M* =0 .40, *SD* =0 .88).

We also examined the simple effect of discrete emotion within each age group to probe the interaction. This indicated a statistically significant effect of discrete emotion among both younger, *F*(2.04, 222) = 116, *p* < .001, *η*^2^ = .46 and older children, *F*(1.85, 184) = 515, *p* < .001, *η*^2^ = .81. Pairwise comparisons revealed that happiness was reported more than anger, embarrassment, fear, or sadness in both age groups (*t*s > 10.54, *p*s < .001, *d*s > 1.01). Fear did not differ from embarrassment for either age group, but older children reported experiencing more embarrassment (*M* = 0.20, *SD* = 0.43) than anger (*M* =0 .02, *SD* = 0.14), *t*(99) =  − 3.93, *p* =0 .003, *d* =  −0 .39, 95% CI [−0 .55, − 0.23]. Among younger children, there were significant differences in ratings of anger and sadness such that they reported experiencing more intense sadness (*M* =0 .39, *SD* =0 .88) than anger (*M* = 0.02, SD = 0.13), *t*(109) =  − 4.36,* p* < .001, *d* =0 .42, 95% CI [−0 .53, −0 .31]. Younger children also reported experiencing more sadness (*M* =0 .39, *SD* =0 .88) than fear (*M* =0 .09, *SD* =0 .44), *t*(109) =  − 3.15, *p* <.04, *d* =  −0 .30, 95% CI [−0 .47, −0 .13].

In line with our predictions and consistent with the patterns reported above for which emotions are endorsed as better to show to others versus hide, children consistently self-reported experiencing more intense positive than negative emotions throughout the study. In contrast to our predictions, however, younger children reported experiencing significantly more sadness than older children, but not more anger, fear, or embarrassment. There were also interesting differences between specific negative emotions, with both younger and older children reporting anger as the least intensely experienced of the five discrete emotions; anger was reported as less intense than embarrassment (for the older children), and less intense than sadness and fear (for the younger children).

### Affective Behavior

**Fig. 3:**
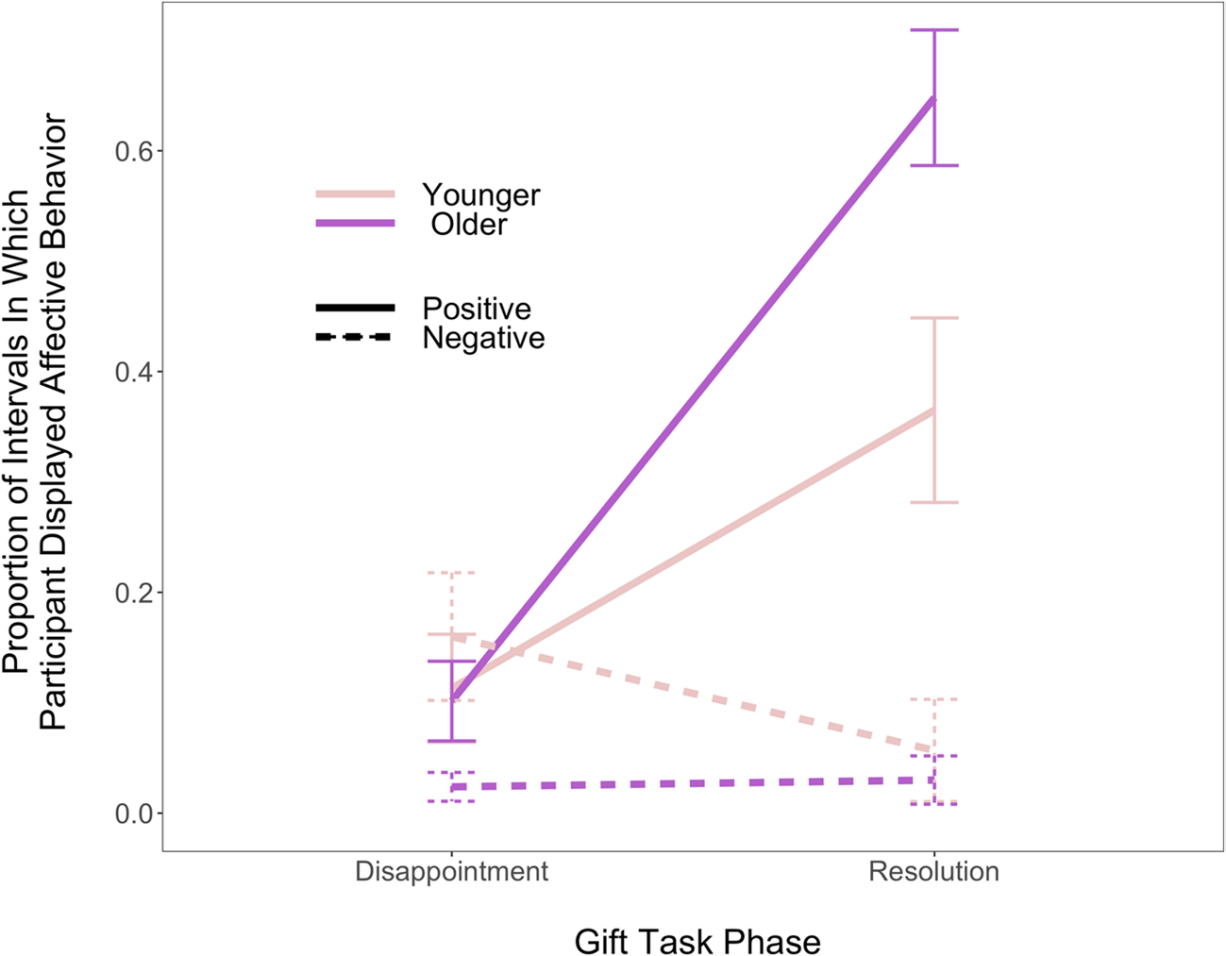
Interaction of age group and gift task phase predicting the proportion of intervals in which participants displayed affective behavior. Error bars indicate standard error. Children displayed more positive than negative affective behavior across both phases of the task and displayed more affective behavior overall during the resolution phase. Older children displayed more affective behavior during the resolution phase than the disappointment phase, although the proportion of younger children’s affective behavior was not significantly different between the two task phases. Finally, older children displayed fewer negative than positive affective behaviors, whereas there was no significant difference in the proportion of negative versus positive affective behaviors displayed for younger children

To assess whether children would display more positive than negative affective behaviors overall and whether there would be age differences in this pattern, we conducted a mixed factorial ANOVA to evaluate the between-subject effect of age group (younger versus older) and within-subjects effects of affect valence (positive versus negative) and phase (disappointment versus resolution) on children’s affective behavior proportions. Results indicated significant main effects of affect valence, *F*(1, 40) = 30.02, *p* < .001, *η*^2^ = .21 and phase, *F*(1, 40) = 31.24, *p* < .001, *η*^2^ = .13, but not age group, *F*(1, 40) = .53, *p* = .47. Children demonstrated more extensive positive (*M* =0 .30, *SD* = 0.35) than negative (*M* = 0.07, *SD* = 0.19) affective behaviors overall, and engaged in more affective behavior during the resolution (*M* = 0.27, *SD* =0 .37) than the disappointment phase of the task (*M* =0 .10, *SD* =0 .20). As shown in Fig. 3, there were also significant interactions of affect valence x phase, *F*(1, 40) = 44.50, *p* < .001,* η*^2^ = .19, age group x phase, *F*(1, 40) = 10.36, *p* = .003, *η*^2^ = .05, and age group x affect valence, *F*(1, 40) = 6.18, *p* = .02, *η*^2^ = .05, but no three-way interaction, *F*(1, 40) = 1.90, *p* = .18.

To probe the interaction between affect valence and phase, we explored the simple effect of affect valence at each phase of the task. This indicated a significant difference in the proportion of negative versus positive affective behaviors displayed during the resolution (but not the disappointment) phase, *F*(1, 41) = 45.80, *p* < .001, *η*^2^ = .40. Specifically, during the resolution phase, children displayed significantly greater proportion positive (*M* =0 .50, *SD* = 0.37) than negative (*M* =0 .04, *SD* =0 .17) affective behaviors, *t*(41) =  − 6.77, *p* < .001, *d* =  − 1.04, CI [− 1.64, −0 .64].

To probe the interaction of age group and phase, we explored the simple effect of phase for each age group. This indicated a significant difference in the proportion of affect displayed between the disappointment and resolution phases of the gift task for older, but not younger, children, *F*(1, 39) = 25.70, *p* < .001, *η*^2^ = .20. Specifically, older children displayed more affective behavior during the resolution phase (*M* = 0.34, *SD* = 0.37) than the disappointment phase (*M* =0 .06, *SD* =0 .13), *t*(47.80) =  − 4.44, *p* < .001, *d* =  − .99, CI [− 1.43, −0 .65].

Similarly, when we probed the interaction of age group and valence by examining the simple effect of valence for each age group, the effect was significant only among older children, *F*(1, 39) = 36.50, *p* < .001, *η*^2^ = .32, who displayed fewer negative (*M* =0.03, *SD* = 0.08) than positive (*M* =0 .38, *SD* = 0.36) affective behaviors, *t*(42.90) =  − 6.05, *p* < .001, *d* =  − 1.35, CI [− 1.81, −0 .98].

In line with our hypothesis, results further indicate that children generally behaved in ways that are consistent with Yucatec Maya cultural display rules, exhibiting more extensive positive affective behaviors like smiling and laughing than negative affective behaviors like pouting, crossing arms, or frowning. Additionally, older children reported and displayed significantly more happiness and positive affective behavior than younger children—which aligns with the age-related differences previously reported for whether it is better to show or hide negative versus positive emotion (older children endorsed happiness as better to show to others more often than did younger children).

### Physiology

To investigate effects of age group (younger versus older) and task phase (baseline, disappointment, resolution) on children’s physiology, we ran two mixed factorial ANOVAs, one for RSA (parasympathetic activity) and one for PEP (sympathetic activity).

**Fig. 4:**
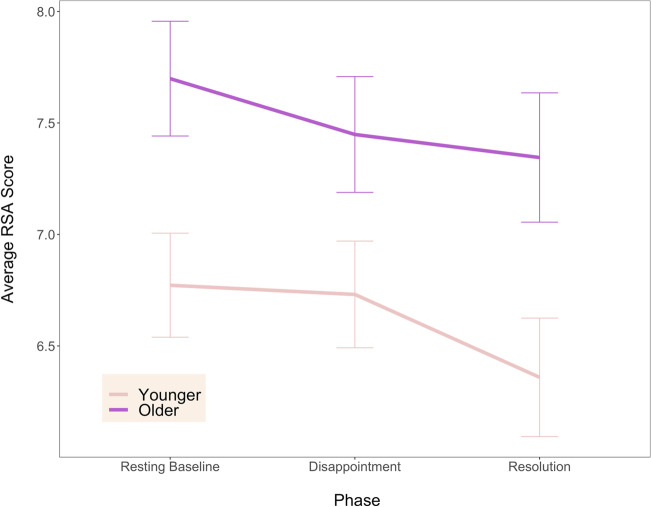
Average RSA (respiratory sinus arrythmia) score for younger and older children across resting baseline, disappointment, and resolution phases of the gift task. Error bars indicate standard error. Older children had significantly higher RSA than younger children across all three task phases. RSA for both age groups was significantly *lower* during the resolution phase than the baseline phase

**Fig. 5:**
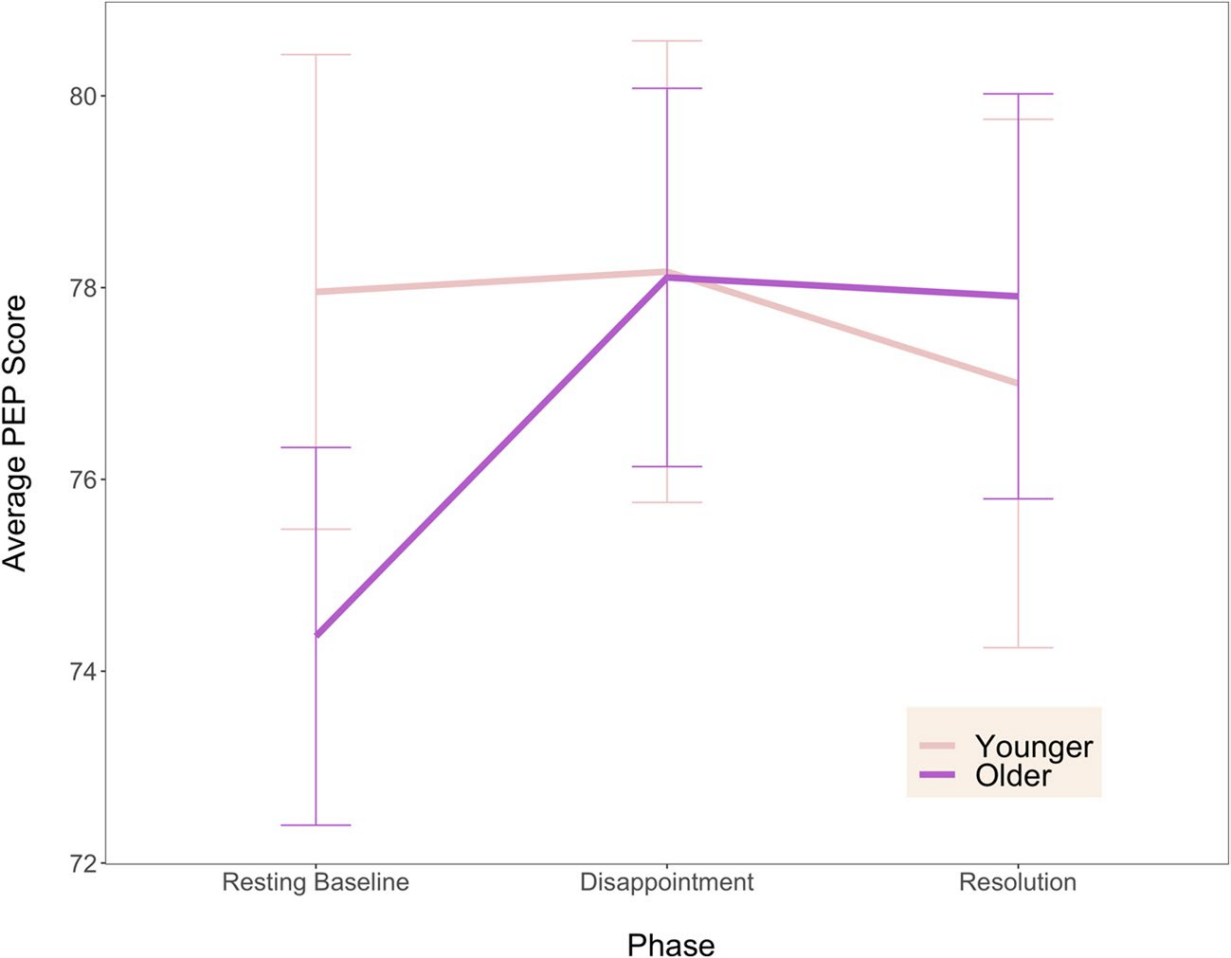
Average PEP (pre-ejection period) score for younger and older children across resting baseline, disappointment, and resolution phases of the gift task. Shorter PEP is indicative of greater sympathetic activation. Error bars indicate standard error. There were no significant effects of age, phase, or their interaction

Results for the RSA model (see Fig. 4) indicated a significant main effect of age group, *F*(1, 39) = 6.38, *p* = .02, *η*^2^ = .13 such that older children (*M* = 7.46, *SD* = 1.11) showed greater parasympathetic activity than younger children (*M* = 6.62, *SD* = 1.15) overall. There was also a main effect of phase, *F*(1, 78) = 6.23, *p* = .003, *η*^2^ = .02, and pairwise comparisons between the three phases indicated that RSA was significantly *lower* during the resolution phase (*M* = 6.80, *SD* = 1.30) than the baseline phase (*M* = 7.19, *SD* = 1.16), *t*(40) = 3.73, *p* = .002, *d* = 0.58, CI [0.30, 0.90]. There were no other pairwise phase differences, and there was no interaction between age group and time point, *F*(2, 78) = .71, *p* = .49. The PEP model (see Fig. 5) showed no significant effects of age, phase, or their interaction (*F*s < 2.67, *p*s > .09). These results did not support our hypotheses, as we did not expect to observe decrements in RSA from baseline to the resolution phase, nor for the PEP levels to remain stable across phases.

## Discussion

This study aimed to contribute to the pursuit of more diverse and equitable representation in study samples, motivate the use of more concurrent multimethod approaches to studying emotion, and explore how emotional processes develop, particularly in the context of cultures where display rules are different from what have been previously studied. These aims epitomize the future of affective science as a field implementing cutting-edge methods in concert with careful attention to cultural nuance. We examined an understudied majority-world population to elucidate how cultural display rules may influence children’s understanding, experience, and expression of emotions.

Previous work suggests that Yucatec Maya adults view negative emotions as important to hide while happiness is considered to be the baseline state of normal functioning (Hanks, 1993; Le Guen, 2017, 2018). We assessed to what extent children conform to these cultural display rules. As hypothesized, across indices of self-report, observational, and physiological measures, we found converging evidence that children would demonstrate knowledge of and adherence to their culture’s display rules—with older children in greater concordance with those display rules. Children’s self-reported experiences and observed expressions also aligned with the idea that positive affective experiences are more acceptable to openly report and display than negative ones, with older children showing a more pronounced version of this pattern, again highlighting the importance of considering developmental progression toward full mastery of these expectations. The timing of these self-reported experiences, however, were not yoked to the timing of emotion-eliciting events or tasks (e.g., we did not collect self-reported experience immediately after receiving the disappointing gift), which may have contributed to the overall pattern of reporting happiness significantly more than other emotions.

When presented with the forced-choice between whether the five discrete emotions of sadness, anger, fear, embarrassment, and happiness were okay to show to others versus should be hidden, older children endorsed happiness as okay to show to others significantly above chance levels and a significantly greater proportion of them endorsed happiness as okay to show compared to the negative emotions. Younger children did not show any significant patterns regarding their self-reported beliefs about showing versus hiding the five emotions. The age difference found between older and younger age groups regarding the appropriateness of showing happiness suggests that while younger Yucatec Maya children may have yet to fully internalize the cultural norms and are perhaps “overgeneralizing” the rules about hiding negative emotions to *all* emotions, older children have acquired a more sophisticated and clear understanding of specific emotion contexts such that happiness stands apart from the negative experiences.

Finally, we obtained some unexpected patterns of physiological responding. While RSA was consistent from baseline to the disappointing portion of the gift task, potentially indicating sustained regulation efforts (parasympathetic maintenance), it was lower than baseline levels during the resolution phase. This decrease in RSA may indicate that children discontinued regulation efforts to enable more natural engagement with the (likely positive) emotions experienced during that phase. Regarding sympathetic nervous system activation, we found no significant change in PEP across the study phases, indicating relative stability in sympathetic arousal rather than the predicted pattern of SNS increases in response to the emotional challenge. The specificity of physiological reactivity to the parasympathetic index suggests that ANS physiology in this study provides more insight into unobservable aspects of emotion *regulation* than *arousal.*

Our findings illustrate that cultural norms inform children’s emotional responding. Maya children as young as six responded in accord with display rules when asked about norms for emotion expression, as well as when confronted with an in vivo emotional challenge. Because Yucatec Maya believe that the primary driver of development is maturation rather than experience, adults rarely scaffold children’s behaviors via explicit instruction (Gaskins, 1996, 2006; Rogoff et al., 1993). Rather, children learn primarily via observation. Thus, affective and developmental science would benefit from additional mapping of trajectories of emotional development in cultures, like the Maya, that use less child-directed input.

We emphasize that emotions are grounded in the sociocultural context in which they occur (Markus & Hamedani, 2010). Despite the (largely negative) consequences of expressive suppression for Western populations (e.g., Butler et al., 2003; Gross & Levenson, 1993, 1997), for East Asian cultures in which expressive suppression is more normative, expected, and valued, the act of hiding emotions aligns with cultural mandates to preserve social harmony, attenuating suppression’s negative consequences (Gross & Cassidy, 2019; Soto et al., 2011; Yeh et al., 2017). The indigenous Yucatec Maya are currently underrepresented in the published literature and have display rules about negative emotion that parallel East Asian cultures (but that are formed within different environmental, developmental, and socialization contexts). Thus, our findings provide an important contrast to existing comparisons made between similarly educated, industrialized, and rich cultures (e.g., North American versus East Asian).

### Limitations and Future Directions

While this study represents an important first step toward a more globally inclusive and robust field of affective science, several limitations bear mention. By acquiring physiological indices in conjunction with observable behaviors and self-reported experience, we were able to explore observable *and* unobservable components of emotional responding. Time and resource constraints, however, precluded us from directly testing the mechanisms by which community members’ beliefs and values about emotions are explicitly or implicitly transmitted to children. Future work with this and other indigenous populations could assess family members’ emotional functioning and beliefs to further characterize the patterns of emotional responding children show under conditions of challenge. For example, information about adults’ ethnotheories of emotion, beliefs and practices surrounding emotion socialization and child development, and observations of how display rules are communicated to youth would all provide important context. In addition, while the current study asked children about whether each of the discrete emotions were okay to show versus should be hidden, this approach requires us to speculate about the reasons for their response choices. While it is possible that children are reporting based on their understanding of Maya cultural norms about suppression and expression of emotions, future work may benefit from asking children to elaborate on *why* they think certain emotions are okay to show versus should be hidden, giving them the option to say that they are unsure or do not know, and asking questions about cultural norms beyond those pertaining to emotional expression to continue to inform research in this area.

Our challenge task leveraged the classic disappointing gift paradigm in a new population and evoked mild distress from unexpectedly receiving an undesirable prize. This, however, limited the specific emotions we could examine; a broader exploration of discrete emotional responding would be fruitful. A related limitation concerns the fact that the disappointing gift paradigm was developed by Western researchers (specifically, for North American samples). Future research with indigenous populations could take a more emic approach to creating emotional challenge tasks, to ensure maximal relevance of the paradigm. These suggestions for future work are not specific to the study of the Yucatec Maya but broadly relevant for investigations into emotional development and affective functioning of cultural groups across the globe and ages across the lifespan. Finally, we deliberately recruited participants from two age groups to examine younger and older children’s emotional responding, but future studies would benefit from longitudinal data collection across a wider age range and with more participants, to elucidate whether and when a shift in understanding of cultural norms surrounding the expression or suppression of emotion occurs.

## Conclusion

The future of affective science lies in the pursuit of more diverse and equitable representation in study samples, use of multimethod approaches to studying emotion, and increased exploration of how emotional process develop. This is the first empirical study to begin to address all of these goals by examining emotional development among the indigenous Yucatec Maya of Mexico. Our findings contribute to knowledge about children's emotional development beyond Western, English-speaking, industrialized/economically developed contexts.

## Footnotes

1 The term “majority-world” is used to refer to societies in Asia, Africa, Latin America, and the Caribbean—because these are the areas where the majority of humans live (Kagitcibasi, 2002; Thalmayer et al., 2021).

2 Minimum wage in the USA in 2018 and 2019 was USD $7.25/h.

3 On rare occasions, Yucatec women may also work outside the village as nannies or au pairs.

4 On two occasions, Yucatec participants arrived with an older sibling acting as their chaperone, in which case the sibling remained in the room with the child, seated behind them and out of view.

5 A Spanish term was used for sadness (triiste) because the Yucatec Maya language does not have a word to describe this discrete emotion at low-to-moderate intensity.

6 The self-report rating after the gift task was collected after the resolution phase.

7 This somewhat conservative range was chosen to fall between the recommended range used for early childhood (.24–1.04 Hz; Bar‐Haim et al., 2000) and adults (.12–.40 Hz; Porges, 1986). Adjusting these parameters to fall between the early childhood and adult ranges has been used in previous studies with wide age ranges like this one (Porges et al., 2013; Quiñones‐Camacho & Davis, 2018).

## Additional Information

### Data Availability

The datasets generated during and/or analyzed during the current study are available in the OSF repository, 10.17605/OSF.IO/29HSX.
